# Cannabinoids for SARS-CoV-2 and is there evidence of their therapeutic efficacy?

**DOI:** 10.3906/biy-2105-73

**Published:** 2021-08-30

**Authors:** Ahmet ONAY, Abdulselam ERTAŞ, Veysel SÜZERER, İsmail YENER, Mustafa Abdullah YILMAZ, Emine AYAZ-TİLKAT, Remzi EKİNCİ, Nesrin BOZHAN, Sevgi İRTEGÜN-KANDEMİR

**Affiliations:** 1 Department of Biology, Faculty of Science, Dicle University, Diyarbakır Turkey; 2 Department of Pharmacognosy, Faculty of Pharmacy, Dicle University, Diyarbakır Turkey; 3 Department of Pharmacy Services, Vocational School of Health, Bingöl University, Bingöl Turkey; 4 Department of Analytical Chemistry, Faculty of Pharmacy, Dicle University, Diyarbakır Turkey; 5 Dicle University Science and Technology Research and Application Center, Diyarbakır Turkey; 6 Department of Biology, Faculty of Science and Literature, Batman University, Batman Turkey; 7 Department of Field Crops, Faculty of Agriculture, Dicle University, Diyarbakır Turkey; 8 Department of Medical Biology, Faculty of Medicine, Dicle University, Diyarbakır Turkey

**Keywords:** Antiviral, cannabidiol, cannabinoids, cannabinoid system, COVID-19, SARS-CoV-2

## Abstract

To combat the coronaviruses and their novel variants, therapeutic drugs and the development of vaccines that are to be effective throughout human life are urgently needed. The endocannabinoid system (ECS) acts as a modulator in the activation of the microcirculation, immune system, and autonomic nervous system, along with controlling pharmacological functions such as emotional responses, homeostasis, motor functions, cognition, and motivation. The ECS contains endogenous cannabinoids, cannabinoid receptor (CBRs), and enzymes that regulate their biosynthesis, transport, and degradation. Moreover, phytocannabinoids and synthetic cannabinoids that mimic the action of endocannabinoids also play an essential role in the modulation of the ECS. Cannabinoids, the main constituents of cannabis (*Cannabis sativa* L.), are therapeutic compounds that have received international attention in the health field due to their therapeutic properties. Recently, they have been tested for the treatment of COVID-19 due to their antiviral properties. Indeed, cannabinoid-type compounds, and in particular cannabidiol (CBD), isolated from glandular trichomes found in the calyx of cannabis flowers with reported antiviral properties is hypothesized to be a therapeutic option in the ministration of SARS-CoV-2 consorted with COVID-19 disease. The relevant articles were determined from the database search published mainly in Web of Science, Google scholar, PubMed, Crossref, and ClinicalTrials.gov database during the pandemic period. The articles were evaluated for the therapeutic potentials, mechanisms of action of cannabinoids, the roles of the ECS in the immune system, impact of cannabinoids in SARS-CoV-2 septic, especially if they address the application of cannabinoids as drugs for the curability and management of SARS-CoV-2 and its novel variants. Although the evidence needed to be considered using cannabinoids in the control and treatment of viral diseases is currently in its infancy, they already offer an opportunity for clinicians due to their effects in relieving pain, improving appetite, and improving childhood epilepsy, especially in cancer and human immunodeficiency virus (HIV/AIDS) patients. In addition to these, the most recent scientific evidence emphasizes their use in the treatment of the coronavirus infected patients. In brief, all preclinic and clinic studies that have been reported show that, through the cannabinoid system, cannabinoids, particularly CBD, have many mechanisms that are effective in the treatment of patients infected by SARS-CoV-2. Thus, more extensive studies are necessary in this area to fully identify the effects of cannabinoids on SARS-CoV-2.

## 1. Introduction

To date, seven different strains of coronavirus have been identified to have infected humans (Liu et al., 2021). These include HCoV-OC43 (OC43), HCoV-NL63 (NL63), HCoV-HKU1 (HKU1), HCoV-229E (229E), Middle East respiratory syndrome coronavirus (MERS-CoV), severe acute respiratory syndrome coronavirus (SARS-CoV), and SARS-CoV-2. Being an infectious disease, COVID-19 is caused by human infection with SARS-CoV-2 (Desai and Patel, 2020). It is usually characterized by inflammatory response manifested by proinflammatory cytokine production, overexpression of C-reactive protein (CRP), neutrophil count, higher TNF, blood urea, and D-dimer (Conti et al., 2020). Although not as bad as the flu epidemic of 1918, COVID-19 has been the most devastating pandemic among all other horrible outbreaks, such as pandemic influenza occured in 1957–1958 and 1968–1970, swine flu in 2009, SARS in 2002–2004, and MERS in 2012, 2015 and 2018. Some human coronaviruses such as OC43, 229E, HKU1, and NL63 typically cause mild symptoms including an upper tract of the respiratory system in adults (Yang et al., 2020), while SARS-CoV-2, SARS-CoV, and MERS-CoV cause infections in the lower respiratory tract and give rise to bronchitis or pneumonia (Raj et al., 2021). Among human coronaviruses, SARS-CoV-2, SARS-CoV, and MERS-CoV often induce lung cell damage as these coronaviruses develop evolutionarily drug resistance by producing various proteins, which let them escape from the immune system of the host (Molaei et al., 2020). According to the latest data, this immune dysregulation that develops as a result of the attacks of these proteins on cells may be involved in an immunosuppression phase following the pro-inflammatory (cytokine storm) phase, accompanied by a high risk of secondary bacterial infection and peripheral lymphopenia (Boechat et al., 2021).

Numerous studies have been and are being conducted by large-scale investments and research institutions by several governments to immunize or cure COVID 19. According to the WHO COVID-19 Dashboard^1^, as of 2 July 2021, it had been reported that more than 182,319,261 million COVID-19 cases, with over 3,954,324 million deaths occurred globally. In order to prevent this destruction faced by humanity, effective vaccines or drugs are urgently needed for COVID-19. Effective antiviral drugs have been developed as of May 15, 2021, and at least seven different vaccines have already been launched worldwide^2^ and additionally at least 1653 listed vaccine candidates are in development (https://www.who.int/emergencies/diseases/novel-coronavirus-2019/covid-19 vaccines). As of 1 July 2021, a total of 2,950,104,812 vaccine doses have been administered. However, the only treatment currently available for COVID-19 patients is supportive (Lucaciu et al., 2021). The treatment options contain antimalarials, antivirals, antibiotics, immunoglobulins, corticosteroids, immunotherapy, anti granulocyte-macrophage colony stimulating factor (anti-GM-CSF), interleukin-6 (IL-6) inhibitors, convalescent, oxygen therapy, plasma, and circulatory support (Lucaciu et al., 2021). 

^1^WHO (2021). Coronavirus Disease (COVID-19) Dashboard [online]. Website https://covid19.who.int/ [accessed 2 July 2021]

^2^WHO (2021). World health organization/news Dashboard [online]. Website https://www.who.int/news [accessed 15 May 2021] 

The currently applied vaccines aim to target SARS-CoV-2 proteins rationally and specifically to suspend the successive multiplication of the virus. However, extremely infectious and globally spread variants such as P.1, B.1.351, B.1.1.7, and B.1.617 B.1.617 of SARS-CoV-2 have been detected in Brazil, South Africa, United Kingdom (UK), and India, respectively (Burki, 2021). While vaccination is critical in preventing severe illness and death, and controlling the pandemic, there are still uncertainties as to how effective current vaccines will be in controlling the pandemic and preventing subsequent deaths. As stressed above, approximately over the last two decades, we have observed at least three severe respiratory illnesses (COVID-19, MERS, and SARS). As coronaviruses evolved from bat-to-human to human coronavirus disease, coronaviruses might potentially reappear in the short run in the form of epidemics or pandemics owing to their capacity to mutate, infect and recombine their different host mechanisms (El-Sayed and Kamel, 2021). While some of the treatments applied are promising, they may also induce many negative side effects such as hypertriglyceridemia and pancreatitis (Morrison et al., 2020). Therefore, it is imperative to discover alternative strategies for effective treatment. One possible strategy is to identify cannabinoids that exhibit beneficial anti-inflammatory and immune-suppressive effects in preclinical models of various chronic inflammatory diseases through the activation of the cannabinoid system and to gain a deep understanding of their mechanisms. Thus, by inhibiting the replication processes of the coronavirus, there is a chance to develop an effective therapeutic strategy. Although the components and structural organization of human ECS and the mechanism of action of its on SARS-CoV-2 have been extensively studied, research in this area is progressing rapidly. 

As is emphasized in this review, there are many lessons to be learned from studies on the effects of cannabinoids on the immune system in SARS-CoV-2 infection via the ECS. Therefore, the goal of this review is to update the effect and mechanism of cannabinoids in the ECS and their applications as medicines for the management of SARS-CoV-2 in preclinical and clinical studies. These include types of cannabinoids that affect the ECS as anti-inflammatory agents, the ECS, and the roles of the ECS in immunity. The review provides a summary of the interaction of cannabinoids with viral diseases, especially SARS CoV-2, the impacts of cannabinoids in SARS-CoV-2 infection, as well as preclinical and clinical evidence of cannabinoid efficacy, and ends with the identification of knowledge gaps.

## 2. Methodology

**Figure 1:**
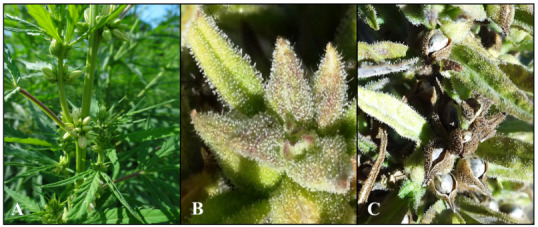
(A) Flowering hermaphrodite hemp plants and (B) glandular trichomes on juvenile or (C) swollen calyx or seeds.

The literature search was comprehensively carried out in search engines such as Google scholar, Web of Science, PubMed, Crossref, and ClinicalTrials.gov database to identify pertinent research articles on COVID-19. Until 21 May 2021, the keywords such as “cannabinoids”, “cannabidiol”, “CBD”, “endocannabinoid system”, “coronavirus”, “SARS-CoV-2 “,” new variants of the SARS-CoV-2”, “COVID-19” were used to solely find the most relevant articles. In general, the most relevant and detailed articles were reviewed, which included information on cannabinoids, cannabinoids as anti-inflammatory agents, the ECS, the role of ECS in the immune system, impact of cannabinoids in SARS-CoV-2 infection, and preclinical and clinic application of cannabinoids as medicines for the control and management of SARS-CoV-2. *C. sativa* L. (hemp or marihuana) is an obligate short-day flowering plant species belonging to the Cannabaceae family and has been used by different cultures for a wide variety of medicinal, psychotropic, and industrial applications for thousands of years (Li, 1974). Natural cannabidiol (CBD) is produced from the glandular trichomes of the cannabis plants. Hemp refers to cannabis plants or materials that contain 0.2% (in the European Union Countries) and 0.3% (in the USA) or less Δ^9^-tetrahydrocannabinol (Δ^9^-THC or simply THC) content, and a relatively high CBD content by dry weight (Small, 1979). In contrast, marijuana or marihuana might be used in place of *C. sativa* plants or materials that contain more than 0.3% THC in its dry weight. *Cannabis* strains with higher CBD, THC or cannabigerol (CBG) content can be bred, depending on the type of cannabinoid to be produced. Figure 1A presents flowering hermaphrodite hemp plants, whereas Figure 1B presents trichome development on the juvenile calyx and Figure 1C mature calyx of female plant inflorescence, respectively.

## 3. Results and discussion

### 3.1. Cannabis L. and Cannabinoids 

*C. sativa* L. is arguably the world’s oldest drug, which has medicinally been used for many millennia in Asian countries since ancient times (Li, 1974). As many as 550 metabolites have already been identified in different strains of *Cannabis* L., including cannabinoids (~150) and >400 non-cannabinoid compounds such as terpenes, hydrocarbons, sugars, nitrogenous compounds, flavonoids, steroids, and amino acids (Paland et al., 2021; Schlag et al., 2021). In addition to a myriad of secondary metabolites, a naturally occurring metabolite, cannabinoids, which have not been detected yet in other plants, are present in the trichomes growing in the leaves and calyxes of *C. sativa* plants (see Figures 1A–1C). Cannabinoids are defined as lipophilic substances acting as ligands for specific types of membrane receptors [commonly called cannabinoid receptor 1 (CB1) and cannabinoid receptor 2 (CB2)] (Sledzinski et al., 2021). Furthermore, these receptors are related to the GPCR (G-protein-coupled receptor) family and form part of the endocannabinoid system.

### 3.2. Types of cannabinoids as anti-inflammatory agents

Cannabinoids are a group of biologically active compounds that have the potential to activate the CB1 found largely and CB2 cannabinoid receptors in the body (Nagarkatti et al., 2020). There are three main types of cannabinoids that affect the ECS: (1) phytocannabinoids, (2) drugs containing synthetic or natural cannabinoids, and (3) endocannabinoids (i.e. anandamide) in animal or human (Pertwee et al., 2010; Apostu et al., 2019; Petrescu et al., 2020).

### 3.2.1. Phytocannabinoids

Phytocannabinoids are naturally present in the cannabis plant. Of more than 150 different cannabinoids reported in cannabis (Schlag et al., 2021), being the main psychoactive ingredient of cannabis, THC was first isolated by Gaoni and Mechoulam (1964), allowing new studies to investigate cannabinoids from multiple perspectives. Being a non-psychoactive compound, CBD is the next most abundant phytocannabinoid to be found in *Cannabis* (Mechoulam et al., 2007). The other extensively studied phytocannabinoids are CBG, cannabinol (CBN), cannabichromene (CBC), Δ^9^-tetrahydrocannabivarin (Δ^9^-THCV or THCV), and cannabidivarin (CBDV). Among the known phytocannabinoids, THC, CBN, CBG, and THCV are the primary psychoactive cannabinoids. They mimic by binding to small molecular receptor molecules embedded in the membranous surfaces of functionally similar molecule cells of the human body (Pertwee et al., 2010). The use of high levels of THC or other psychoactive cannabinoids for recreational or therapeutic purposes creates a state of euphoria as well as anti-inflammatory and analgesic effects, so the medical use of cannabinoids with psychoactive properties has been limited (Dujourdy and Besacier, 2017). The non-psychoactive cannabinoids found in cannabis are CBD, CBC, CBDV, and CBG. While these show low affinity for cannabinoid receptors, they are known to interact with other receptors such as peroxisome proliferator-activated receptors (PPARs, particularly PPARα and PPARγ), the transient receptor potential cation channel subfamily V member 1 (TRPV1), and the orphan G protein-coupled receptors (GPR55, GPR119) (Sledzinski et al., 2021).

Many cannabinoids, particularly CBD, have been proven to act as powerful anti-inflammatory agents in recent studies (Almogi-Hazan and Or, 2020; Nagarkatti et al., 2020; Lima et al., 2021; Nguyen et al., 2021). The Food and Drug Administration (FDA) has approved cannabidiol (Epidiolex) and three synthetic drugs: Cesamet (nabilone), Marinol (dronabinol), and Syndros (dronabinol). Epidiolex consists of a purified form of hemp-derived CBD used to treat patients with seizures correlated to Dravet or Lennox-Gastaut syndromes in patients one year of age and older (Nagarkatti et al., 2020). Most recently, CBD and 7-OH-CBD (a more active metabolite than CBD) have been proposed as potential therapeutic and preventive agents in the early stage of infection with SARS-CoV-2 (Nguyen et al., 2021). CBD, the most studied cannabinoid, might also inhibit the production of proinflammatory cytokines such as interferon gamma, tumor necrosis factor alpha (TNF-α), inducible protein-10-interleukin IL-2, IL-1α and β, IL-6, monocyte chemoattractant protein-1α, macrophage inflammatory protein-1α (Nichols and Kaplan, 2020). Another cannabinoid that has been studied extensively, THC slows proinflammatory IL-17 secretion and proliferation of activated lymphocytes and may increase anti-inflammatory IL-10 secretion (Khuja et al., 2018). In addition, in cell experiments investigating the effect of THC on antibody formation, it has been shown to induce immunosuppression in B cells (Eisenstein et al., 2007). In many animal model studies, THC lessened signaling proteins such as interferon-gamma (IFN-g) and IFN-α pro-inflammatory cytokines (Rossi et al., 2020; Mohammed et al., 2020). Studies have reported that the reduction of TNF-α level occurs as a result of activation of CB1 and CB2 receptors (Nichols and Kaplan, 2020; Costiniuk and Jenabian, 2020). Moreover, CBG is a precursor molecule for major phytocannabinoids such as THC and CBD. CBG has been shown to have therapeutic potential in the treatment of inflammatory bowel disease, Huntington’s disease, neurological disorders such as Parkinson’s disease, Alzheimer’s disease, multiple sclerosis, and epilepsy (Nachnani et al., 2021). Overall, phytocannabinoids have the potential to suppress cytokine storm by acting on cells in different systems in different ways through the endocannabinoid system to suppress inflammation.

### 3.2.2. Drugs and synthetic cannabinoids

FDA approved one cannabis-derived drug that contains natural cannabinoids such as THC and/or cannabidiol represented by Epidiolex or Sativex (Apostu et al., 2019). Since the discovery of THC in 1964, and the recognition of cannabinoids’ therapeutic potential, extensive research has been carried out to produce synthetic cannabinoids (SCBs) that mimic the effects of natural THC (Mills et al., 2015). SCBs, cannabinoid receptor ligands produced by chemical synthesis, have a large family of molecules that mimic the functions of natural cannabinoids. They are used in studies aimed at determining the relationships between the structure and activities of cannabinoids and for therapeutic purposes in medicine, as well as for recreational purposes (Lauritsen and Rosenberg, 2016). They include SR144528, WIN-55,212-2, HU-331, HU-210, JWH-018, JWH-133, and UR-144, but more than 140 are classified in this group (Almada et al., 2020). They are available under four groups: fatty acid amides, aminoalkylindoles, classical cannabinoids, and non-classical cannabinoids (Cohen and Weinstein, 2018). Such analogous cannabinoids are also referred to as cannabimimetic cannabinoids and synthetic cannabinoids. Synthetic cannabinoids, both drunk and eaten, have been commercially marketed for many years. The synthetic cannabinoids that are easily accessible commercially today are as follows: Syndros (dronabinol), Marinol, (Dronabinol), Cesamet (nabilone), Rimonabant, and Zimulti (Apostu et al., 2019). However, the increase in recreational use of SCBs and their therapeutic use may result in tachycardia, breathing disorders, and seizures (Almada et al., 2020). Of the SCBs currently marketed, nabilone is a THC analogue, and dronabinol is a biochemically identical form of THC. Both can be prescribed clinically (Ebbert et al., 2018). By activating CB1 or CB2 receptors, these cannabinoids modulate systems such as the central nervous system, immune, cardiovascular, pulmonary, musculoskeletal, and digestive systems (Apostu et al., 2019).

### 3.3. Endocannabinoid system

The ECS consists of cannabinoid receptors (CBRs), endogenous cannabinoids, and enzymes that take part in their biosynthesis, transport, and degradation (Lu and Mackie, 2016). This system is involved in all of the human body’s internal interactions, including the components of the immune system such as antibodies, white blood cells, the spleen, the thymus, the bone marrow, and the lymphatic system (Apostu et al., 2019).

**Figure 2:**
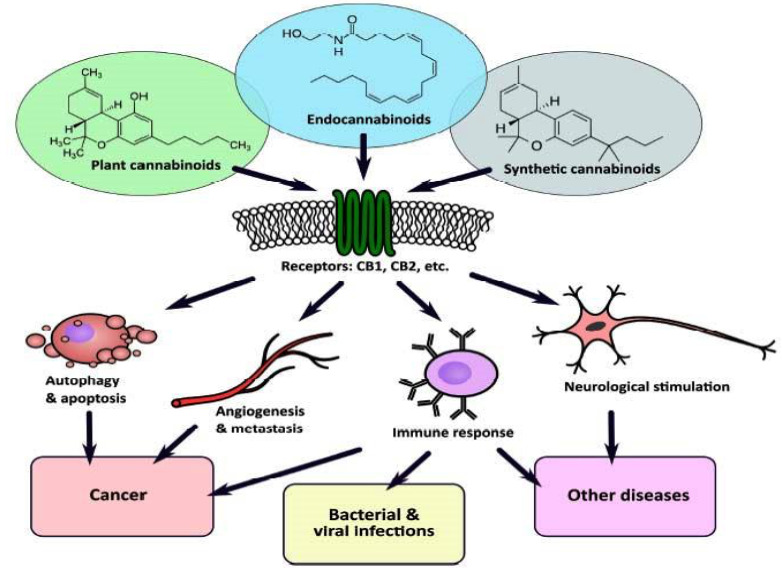
The involvement of the endocannabinoid system in various modulating processes makes it a promising target in the treatment of several disorders including viral infections (Sledzinski et al., 2021).

A summary of the disorders, including viral infections, cancer, and other diseases that are thought to support the treatment of cannabinoids through the cannabinoid system is given in Figure 2 (Sledzinski et al., 2021).

Noticeable changes in the ECS activity have been monitored in pathological conditions including neurological disorders, cancers, and other diseases such as mood/behavior, pain/insomnia, and gastrointestinal disorders (Soliman, et al., 2021; Sledzinski et al., 2021). Therefore, today pharmaceutical modulation of the ECS has been an effective therapeutic strategy, for example by the administration of cannabinoids in the treatment of palliative care and weight loss (Sledzinski et al., 2021). Additionally, the effect of cannabinoid uptake on infectious conditions has been questioned for several years due to its involvement in the immune function of the endocannabinoid system but is of particular interest today in the COVID 19 pandemic (El Biali et al., 2020). Three main component groups have been identified in the modulation of the ECS in the human brain.

### 3.4. Endocannabinoids (endogenous cannabinoids)

So far, in mouse and human model studies, at least two main endocannabinoids (eCBs), 2-Arachidonyl Glycerol (2-AG) (Devane et al., 1992) and N-arachidonyl ethanolamine (Anandamide, AEA) have been identified (Stella et al., 1997). They are metabolized very rapidly by enzymes such as monoacylglycerol lipase (MAGL) and fatty acid amide hydrolase (FAAH) for 2-AG and anandamide (AEA), respectively (Shamran et al., 2017). They are responsible for providing signaling with cannabinoid receptors (Iannotti and Vitale, 2021). The degradation pathways and synthesis of AEA and 2-AG are almost completely different; although both are derived from arachidonic acid, different enzymes mediate their synthesis (Sledzinski, 2021). Different biosynthesis methods have been described for the synthesis of AEA; hydrolysis of NAPE (N-acylphosphatidylethanolamine) with NAPE selective phospholipase D is the most known biosynthesis method (Kumar et al., 2019). Elevated levels of 2-AG and AEA are especially found in the corpus striatum and brainstem (Greenberg, 2003). In addition to these two main cannabinoids, four putative endocannabinoid ligands, (1) O-arachidonoyl ethanolamine (virodhamine) (Porter et al., 2002), (2) noladin ether (2-arachidonoyl glyceryl ether, 2-AGE) (Hanus et al., 2001), (3) NADA (N-arachidonoyl dopamine) (Huang et al., 2002) and (4) oleic acid amide (oleamide, OA) (Laezza et al., 2020) have also been identified. Excessive alcohol use can trigger the overproduction of endocannabinoids and affect cardiovascular function by CB1 receptor signaling (Paloczi et al., 2019). The neutral arachidonate derivative, 2-AG is one of the main sources of arachidonic acid in the synthesis of prostaglandins and plays a role in the metabolism of lipids (Baggelaar et al., 2018). Brain prostaglandins that promote neuroinflammation are formed as a result of hydrolysis of endocannabinoids (Nomura et al., 2011). Nevertheless, hydrolysis of 2-arachidonic acid by phospholipase C (PLC) and diacylglycerol lipase α or β (DAGL α or DAGL β) produces 2-AG (Kumar et al., 2019).

### 3.5. Cannabinoid receptors

G-protein-coupled receptors (GPCRs) and transient receptor potential channels (TRPs ), which are embedded in the cell membrane, have been determined as cannabinoid receptors (Paland et al., 2021; Rohbeck et al., 2021). The receptors CB1, CB2, GPCR18, and GPCR55 are members of the GPCRs family (Almogi-Hazan and Or, 2020). The human body has thousands of GPCRs. These include dopamine, opioid, serotonin, and adrenergic receptors (Small, 1979). TRPV1-4, TRPA1, and TRPM8 are TRPs that are supposed to be cannabinoid receptors (Storozhuk and Zholos, 2018). TRP channels regulate numerous neural signaling processes and physiological roles such as smell, pain perception, taste, vision, temperature sensation, or pressure sensing (Moran et al., 2011). Molecules binding to cellular receptors are chemically called ligands. Pharmacologically, agonists are defined as the chemicals that contact and activate receptors (Pertwee, 2010). Both AEA and 2-AG are agonists at CB2 and CB1 receptors. In general, many antagonists show high selectivity for the CB1 receptor, allowing differentiation between CB1 and CB2, while a large number of agonists show low selectivity between cannabinoid receptors. However, some agonists, such as the arachidonyl-20-chloroethylamide compound, show high selectivity to CB1 (Howlett and Abood, 2017). Moreover, the ligand (molecules that bind to cellular receptors) selectivity, crystal structures, and functions of these receptors have recently been determined (Li et al., 2019). 

**Figure 3:**
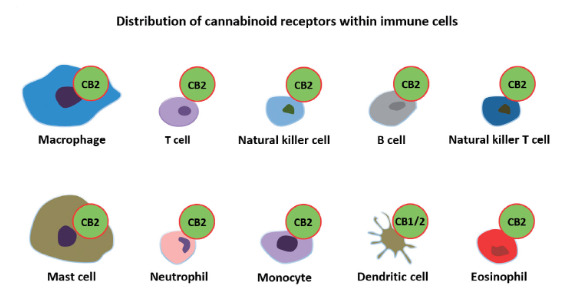
Cannabinoid receptors in immune cells (Lucaciu et al., 2021).

Cannabinoid receptors are the most common type of GPCR in the brain. The CB1 receptor is expressed predominantly in the central nervous system (CNS) and various non-neural peripheral tissues, including the intestine and vasculature, particularly in neuromodulatory roles, whereas the CB2 receptors that are expressed in the spleen and lymph nodes are known for modulating the immune response and inflammation (Rossi et al., 2021; Lucaciu et al., 2021; Figure 3). CB2 receptors in the immune system’s cells are present in T4 lymphocytes, B lymphocytes, leukocytes, T8 lymphocytes, macrophages, mononuclear cells, microglia, mast cells, natural killer cells, and in several organs and tissues such as the brain, liver, spleen, tonsils or lymph nodes, thymus, lung, kidney (Cabral and Griffin-Thomas, 2009; Rossi et al., 2020). 

**Figure 4:**
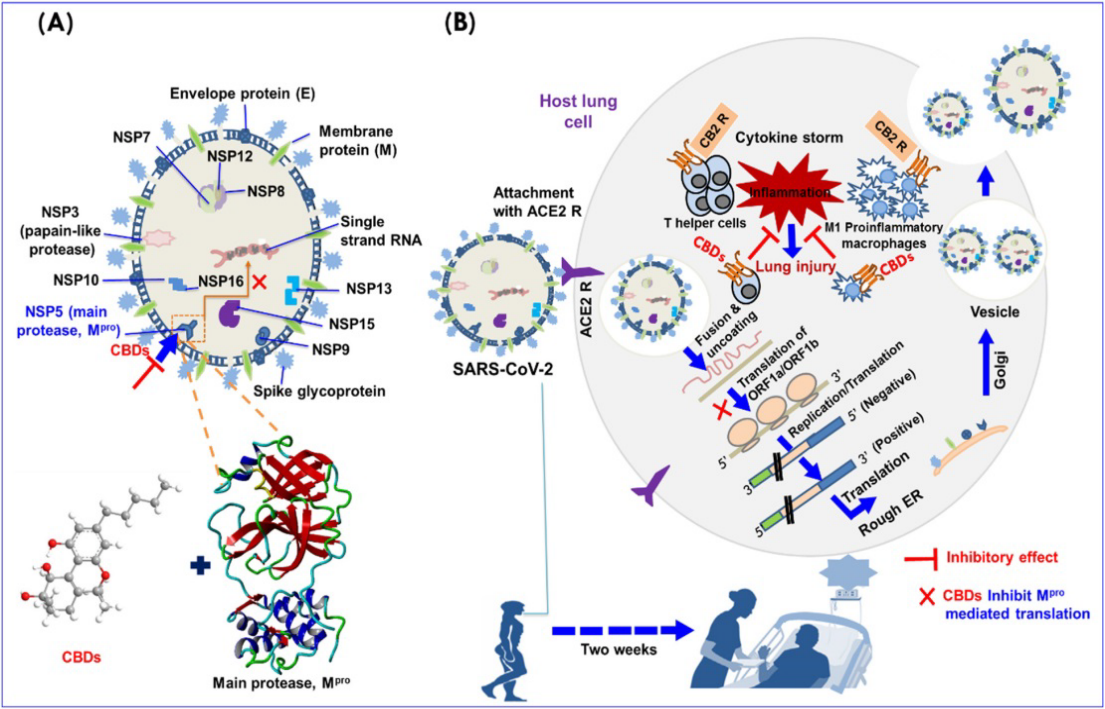
The effect of cannabinoids on the immune system in SARS-CoV-2 infection. A. Structure features of SARS-CoV-2 and its main SARS-CoV-2 Mpro binding pocket, B. SARS-CoV-2 life cycle in host lung cells is initiated by binding of ACE2 cellular receptor to viral spike glycoprotein (Raj et al., 2021).

It is known that stimulation of CB2 receptors improves the immune-modulating properties of mesenchymal stromal cells, limits the release of proinflammatory cytokines, and shifts the macrophage phenotype to the anti-inflammatory M2 type (Rossi et al., 2020). Because of these known functions and as shown in Figure 4, the CB2 receptor should be a therapeutic target in the emergency of the COVID-19 pandemic. Instead, CB1, immensely correlated to the psychoactive effects of cannabinoids, is expressed at low levels in peripheral tissues and mostly in the central nervous system (Farquhar-Smith et al., 2000; Rossi et al., 2020).

In addition to CB2 and CB1 receptors, the ECS has also been described to modulate a large number of candidate receptors (they may be named as CB3 receptor) and channels with the inclusion of sundry TRP (transient receptor potential) channels, GPCR channels such as G-GPR55, a receptor linked with seven transmembrane G proteins, GPR119, GPR18, glycine receptors, -aminobutyric acid (GABA) A, and peroxisome proliferator activated receptors (PPARs) such as PPAR-β /δ, PPAR-γ and PPAR-α or transient receptor potential vanilloid 1 (TRPV1) (Apostu et al., 2019; Ghaffari et al., 2020). Among the CB_3_ candidates, GPR55 has gained much attention for its activation by cannabinoids and its ability to activate the immune system (Yang et al., 2016; Lucaciu et al., 2021). Some phytocannabinoids, particularly THC, mediate their biological effects primarily through CB2 and CB1 receptors. THC might act as an agonist of the channels/receptors GPR18, GPR55, transient TRPV4, TRPV3, TRPV2, TRPA1, PPAR, and as an antagonist of the channels/receptors 5-HT3A and TRPM8 (Martinez et al., 2020). However, CBD may act as an agonist of adenosine channels/receptors TRPV3, TRPV2, TRPV1, TRPA1, PPAR, 5-HT1A, A1, and A2 adenosine, and as an antagonist of 5-HT3A, GPR18, and GPR55 receptors (Burstein, 2015; Olah et al., 2017). In addition, CBD raises AEA levels and is an inverse agonist of the GPR12, GPR6, and GPR3 receptors.

### 3.6. Endocannabinoid enzymes

The enzymes responsible for the inactivation of endocannabinoids (2-AG and AEA) are fatty acid amide hydrolase (FAAH) inhibitors and MAGL, respectively (Egmond et al., 2021).

MAGL and FAAH might implement therapeutic effects without causing unpleasant side effects correlated with direct CB1 receptor stimulation by THC (Egmond et al., 2021). Palmitoyl and oleoyl ethanolamide are some of the many fatty acid amides on which FAAH has a catabolic effect (Mastinu et al., 2018). Therefore, natural or many synthetic molecules that inhibit FAAH can generate biological responses that are not limited to ECS (Kumar, et al., 2019). Endocannabinoids can also undergo oxidative metabolism by cytochrome P450 (Snider et al., 2010), lipoxygenases (Kozak et al., 2002), and cyclooxygenases (COX-2) (Kozak et al., 2000), forming new molecules such as prostamides with potential physiological roles (Alhouayek and Muccioli, 2014). Moreover, alpha/beta domain hydrolases 6 and 12 (ABHD 6 and 12) and COX-2 might also play a role in the catabolism of 2-AG.

### 3.7. The roles of the ECS in immunity

The ECS has anti-inflammatory activities in adaptive and innate immunity (Paland et al., 2021). In general, the ECS functions in many systems in the human body, including the musculoskeletal system, central nervous system, immune system, and gastrointestinal system (Lucaciu et al., 2021). The immune system is defined as a complex system of protein and cell networks, all connected and working together to fight infections. The ECS plays a role in mature immune cell monitoring and regulation of effector cell functions (Almogi-Hazan and Or, 2020). B cells, T cells, macrophages, basophils, dendritic cells, mast cells, eosinophils, neutrophils, natural killer cells, natural killer T cells, CD8^+ ^cells, and CD4^+^ cells are examples of immune system cell types (Figure 2; Lucaciu et al., 2021; Nichols and Kaplan, 2020). Like most cell types in the human body, immune cells also produce cannabinoids called endocannabinoids. These are endogenous ligands for CB1 and CB2 receptors derived from arachidonic acid (Nagarkatti et al., 2020). The response and effects of the immune system take place through signal proteins, i.e. antibodies, which are expressed or secreted proteins called chemokines or cytokines (Nichols and Kaplan, 2020). CB2 deficiency, one of the cannabinoid receptors present in immune cells, as well as in the entire immune system, has been reported to increase acute neutrophil mobilization in areas of inflammation (Kapellos et al., 2019). It has been reported that monocytes/macrophages and microglial cells express CB1 and CB2 receptors in both animals and cellulo models of inflammatory diseases (Turcotte et al., 2016). The role of CB2 in the retention of immature B cells in the bone marrow has been proven by Pereira et al. (2009). In addition, ECS also plays a much more direct role in regulating adaptive immunity, the effect of which is long-lasting. For example, in the experimental autoimmune encephalomyelitis model, CB2-deficient T cells in the central nervous system showed a high proliferation rate, a decreased level of apoptosis, and an increase in inflammatory cytokine production, resulting in severe clinical disease (Maresz et al., 2007). Therefore, a research group has shown that stimulating the immune system is a good approach to prevent viral infections in patients with severe flu (Hui et al., 2018). A recent article has reported that immune system activation by the endocannabinoid system has reduced or stopped viral replications, showing a reduction in virus entry and leading to a reduction in proinflammatory cytokines such as TNFα or IFNg, IL-2, IL-4, IL-6, IL-12 (Lucaciu et al., 2021). A combined therapeutic approach has been suggested to prevent host overreaction to the invasion of SARS CoV-2 (Lucaciu et al., 2021), which is associated with an overreaction of the immune system and a cytokine storm (Tay et al., 2020). It has also been reported that, with respect to SARS-CoV-2 infection, cannabinoids in the immune system have the potential to reduce the mortality rate by limiting the abnormal functions of the immune system (Lucaciu et al., 2021).

The fact that cannabinoid receptors are expressed by immune cells and that these receptor agonists show strong anti-inflammatory activity aiming at cannabinoid receptors demonstrates that the endocannabinoid system is an important regulator of the immune system. Thus, all these studies indicate the need for more intensive research on endocannabinoids as a new approach to the treatment of systemic inflammation, cytokine storm, and acute respiratory distress syndrome (ARDS) in patients with COVID (Nagarkatti et al., 2020).

### 3.8. Cannabinoids and viral illnesses

A viral infection develops as a result of contention between the organism’s adaptive and innate immune system response and the infectious potential of the virus (Sledzinski et al., 2021). The agonists of CB1 receptor such as endocannabinoids can inhibit Ca^2+^ ions release, affecting activation of Ca^2+^-dependent proteins and changing signal transduction (Zou and Kumar, 2018). Malfunction of numerous Ca^2+^ dependent enzyme structures, such as transglutaminases or calpains and matrix metalloproteinases involved in inflammatory processes, can promote virus replication (Reiss, 2010). Several studies have been reported on the antiviral effect of CBD due to its anti-inflammatory properties. Although the antiviral effect of CBD is effective for the treatment of viral hepatitis (Lowe et al., 2017), or influenza (Karmous et al., 2013), HIV (Costiniuk et al., 2019), borna disease virus or vaccinia virus (Tahamtan et al., 2016) and orthopoxvirus, studies on the use of cannabinoids in treating viral diseases caused by all forms of coronavirus, including SARS-CoV-2, are still in their infancy.

In a viral multiple sclerosis model, Nabiximoles improved motor activity as measured by the presence of microglial activity, axonal damage and central nervous system infiltrates, while renovating myelin morphology in a multiple sclerosis viral model (Feliu et al., 2015). In a study of patients living with HIV, cannabis exposure was found to cause lower neurocognitive impairments (Watson et al., 2020). As an antiviral agent, CBD has been shown to have no effect against hepatitis B virus cultured to produce these viruses in cell lines but an antiviral effect against hepatitis C virus (HCV) (Lowe et al., 2017). In another study using a CSHV-infected human dermal microvascular endothelial cell model, CBD has been shown to have an indirect viral effect against Kaposi’s sarcoma-associated herpesvirus (KSHV) (Maor et al., 2012). In another study, CBD was shown to attenuate the effects of neuroinflammation caused by Theiler’s murine encephalomyelitis virus (TMEV) (Mecha et al., 2013). In both HIV and post-Ebola syndrome, CBD has been suggested as a therapeutic agent to control the activation of the immune system (Costiniuk et al., 2019). Dronabinol or THC is approved for the management or treatment of vomiting and weight loss in human immunodeficiency virus (HIV)/acquired immune deficiency syndrome (AIDS) and cancer (Badowski and Yanful, 2018). A recently published study suggested that the antiviral potential of CBD and THC against SARS-CoV-2 is more effective than CBDA, THCA, and CBN, but there may be safety concerns for humans as high doses of CBD or THC cause cytotoxicity in the host cell (Raj et al., 2021). 

### 3.9. Impact of cannabinoids in SARS-CoV-2 infection

COVID-19 is usually characterized by inflammatory response manifested by pro-inflammatory cytokines production (IL-1, IL-6, IL-10), overexpression of C-reactive protein (CRP), neutrophil count, higher TNF, blood urea, and D-dimer (Conti et al., 2020). The spike proteins of the virus bind to ACE2 (angiotensin converting enzyme 2) receptors on the surface of the cell or TMPRSS2-mediated membrane fusion upon ACE2 engagement, and release viral RNA into the cell via endocytosis (Bian and Li, 2021). According to a recent study (Gadanec et al., 2021), ACE2-free intra- and extrapulmonary immune and non-immune cells also demonstrated viral susceptibility. This suggests that the S protein also utilizes toll-like receptors (TLR), C-lectin-type receptors (CLR), the non-immune receptor glucose-regulated protein 78 (GRP78), and neuropilin-1 (NRP1). 

Cannabinoids have the potential to inhibit the secretion of many pro-inflammatory cytokines resulting in the prevention of cytokine release syndromes (CRS) (Paland et al., 2021). Very recently, Raj et al. (2021) reported a preliminary effort to discover dual-acting phytocannabinoids capable of interacting with CB2 receptors in the lungs (agonist) and SARS-CoV-2 M^pro^ as an antagonist. In their computational and in vitro based study, it has been suggested that both CBD and THC can inhibit SARS-CoV-2 in two ways (Raj et al., 2021). They can bind to and inhibit SARS-CoV-2M^pro^ by blocking translation; they also act as agonists of the CB2 receptor, reducing pro-inflammatory cytokine levels in lung cells (Figure 4; Raj et al., 2021). The SARS-CoV-2 genome encodes several proteins (already identified 25 proteins) that the virus needs to infect humans and replicate itself (Parks and Smith, 2020). Among these, SARS-CoV-2M^pro^, the glycoprotein (S), notorious spike (S) protein, which recognizes human ACE2 in the initial stage of infection, chymotrypsin-like main protease, papain-like protease, the RNA polymerase, which synthesizes viral RNA, two proteases, which cleave viral and human proteins, and the RNA-cleaving endoribonuclease are known to play an important role in the progress of SARS-CoV-2 (Parks and Smith, 2020). 

The SARS-CoV-2 life cycle is initiated by binding between the S-protein of SARS-CoV-2 and ACE2 (cellular receptor), a protein with an enzymatically active site on the surface of cells in host lung cells or other organ tissues (Han and Kral, 2020; Zhang et al., 2020a). Spike glycoprotein (S-protein) mediates viral envelope fusion with host cells via endosomal pathways. As a result of the occurring fusion, the viral cell releases the RNA of SARS-CoV-2 into the host cell and converts the viral genome RNA into replicase polyproteins 1ab and pp1a. These proteins are cleaved into small products by proteinases (Romano et al., 2020; Shereen et al. 2020; See Figure 4). Papain-like protease and SARS-CoV-2 M^pro^ are essential for the processing of polyproteins (Zhang et al., 2020b). Later, a sequence of sub-genomic mRNA is formed by the polymerase (Hu et al., 2020). Also, viral proteins and genome RNA are accumulated into virons in the ER and Golgi, and SARS-CoV-2 is transported in vesicles to the extracellular compartment (Raj et al., 2021). During this process, M1 pro-inflammatory macrophages and T-helper cells secrete interleukins released from macrophages and T-lymphocytes, which cause extensive inflammation inside lung cells (Vabret et al., 2020). At this stage, the CB2 receptor activated by CBD administration inhibits inflammatory processes such as macrophage migration into the lungs (Pisanti et al., 2017; Hernandez-Cervantes et al., 2017) and sets therapeutic targets for the reduction of some other immune pathological processes associated with viral infections (Costiniuk and Jenabian, 2020). However, more M2 phenotype macrophages are produced by inhibition of the CB2 receptor, thus causing the production of IL-10 and anti-inflammatory TGF-b (Rossi et al., 2020). Responding to infection with an aggressive inflammatory reaction, the host’s airways are damaged (Wong et al. 2004). As a result, a vast cytokine release occurs by the immune system, causing a cytokine storm associated with typical sepsis symptoms such as breathing problems, abnormal heart function, low platelet count, unconsciousness, and tremors, many of which are associated with fatal COVID cases (Onaivi and Sharma, 2020). Moreover, uncontrolled inflammation affects multiple organs, leading to cardiac, hepatic or renal failures (Lucaciu et al., 2021). 

Discovering drugs that can bind to viral proteins and prevent them from working is a logical route forward and should be a priority for many research laboratories (Parks and Smith, 2020). As SARS-CoV-2 M^pro ^inhibition is unlikely to induce any toxic effects on humans, it is considered the best molecular target for inhibition of coronavirus replication (Zhang et al., 2020a; Al-Khafaji et al., 2020; Abian et al., 2020). As explained in Figure 4 above, by acting as agonists of the CB2 receptor, CBDs have been demonstrated to inhibit SARS-CoV-2 M^pro^ activity and block viral replication due to their binding affinity, complex stability, and in vitro potency (Raj et al., 2021). More importantly, such inhibitors (inhibitor for SARS CoV-2 M^pro^) are unlikely to be toxic, and human protease similar to SARS-CoV-2 M^pro^ has not been reported (Zhang et al., 2020b). Exogenous CBD administration has been shown to suppress inflammatory transcription factors such as AP-1, NF-kB, and NFAT. As a result, cytokines such as IL-6, IL-1b, IL-1a, GM-CSF, and TNFα are suppressed in various cells and tissues (Nichols and Kaplan, 2020). Differentiation of Th17 cells, also shown to be suppressed by CBD, is promoted by IL-6 (Zgair et al., 2017). In murine models of chronic asthma, as a result of CBD administration, cytokine levels of IL-4, IL-5, IL-6, IL-13, and TNFα have been shown to decrease, thereby reducing fibrosis and airway inflammation (Vuolo et al., 2019). Briefly, these anti-inflammatory effects of CBD have been proven to be beneficial in administering CBD to prevent CRS before the inflammatory response becomes pathological in COVID-19 patients.

### 3.10.Preclinical evidence of cannabinoid efficacy

Unfortunately, there are limited publications listed on the effect of CBD on cytokine storm syndrome and acute respiratory distress syndrome associated with COVID-19. Until now, a unified treatment regimen for COVID-19 has not been determined, and most treatments are experimental, meaning that medications used for other syndromes are tested on humans (Yagisawa et al., 2021; Sledzinski et al., 2021). The major cause of the significant morbidity and high mortality rate associated with the disease is the lack of specific treatment for COVID-19 (Egmond et al., 2021). The only treatments available today are represented by supportive care (Song et al. 2020). Treatment options contain antivirals, immunoglobulins, antimalarials, IL-6 inhibitors, corticosteroids, immunotherapy, convalescent plasma, anti-GM-CSF, antibiotics, oxygen therapy, and circulation support (Song et al., 2020; Vijayvargiya et al., 2020).

Recently, a large number of studies have been reported, especially on the possible therapeutic use of cannabinoids for COVID-19. A partial list of published preclinical evidence of cannabinoid efficacy in COVID-19 through some reported observational studies are presented in Table. The first evidence of the effect of cannabis or seed extracts on COVID-19 was reported 3 years ago by a group of Italian researchers (Orio et al., 2017). In their studies, they showed that the four peptide forms, GVLY, IEE, LGV, and RVR, prepared from cannabis seeds, have ACE inhibitory activity. In a recent paper, high-CBD extracts have been reported to down-regulate TMPRSS2 enzymes and ACE2 and crucial viral gateways in oral, lung, and intestinal epithelia constituting important routes of SARS-CoV-2 invasion (Wang et al., 2020). These authors proposed cannabidiol products for mouthwash as a preventive strategy in COVID-19 infection to reduce the entry of SARS-CoV-2 into susceptible hosts by down-regulating the enzymes TMPRSS2 and ACE2. After this study, Esposito et al. (2020) hypothesized that systemic administration of cannabidiol might have the potential to limit the progression of COVID-19 disease and post-infection sequelae because cannabidiol may reduce viral entry by downregulating the TMPRSS2 and ACE2 receptor (Esposito et al., 2020). In line with these studies, some other researchers hypothesized that CBD could be useful as an antiviral (Hill, 2020) or anti-inflammatory (Byrareddy and Mohan, 2020; Costiniuk and Jenabian, 2020) agent for COVID-19. As reported by Huang et al. (2020), the abnormal release of proinflammatory molecules and cytokines are closely associated with lung injury in the SARS-CoV-2 pandemic. Therefore, it is extremely important that antiviral or other compounds used for the treatment of COVID 19 prevent the abnormal release of cytokines and proinflammatory molecules. The effect of natural cannabinoids in reducing ACE2 activity has recently been confirmed (Anil et al., 2021). In that study, CBD, CBG, and THCV fractions were extracted from a *C. sativa* strain and tested in vitro with a standard phytocannabinoid. Both extract fractions of CBD, CBG, and THCV and the standard phytocannabinoid have been found to induce polarization of the macrophage cell line KG1, reduce the secretion of pro-inflammatory cytokines IL-6, IL-8, CCL2, and CCL7 from the alveolar epithelial cell line A549, and increase phagocytosis. In that study, Anil et al. (2021) also reported that the phytocannabinoid formulation containing cannabidiol limits pulmonary fibrosis by decreasing the expression levels of ACE2 and interleukin-7 (IL-7). By decreasing the expression levels of IL-6 and IL-8, the authors suggested that cannabinoid compounds have important anti-inflammatory properties in lung tissue. In another study where viral infections were simulated by using synthetic RNA Poly I: C, it was shown that ARDS induced by Poly I: C could be prevented by CBD (Khodadadi et al., 2020). 

**Table:** A partial list of published preclinical evidence of cannabinoid efficacy in COVID-19 through recently reported studies.

Study type	Tested Cannabinoid	Cannabinoid properties for COVID-19	Findings	Reference
ACE-inhibitory activity test	Peptides extracted from seed such as GVLY, IEE, LGV and RVR	ACE inhibitory activity	Peptide forms extracted from hemp seed had ACE inhibitory activity preventing the entry of SARS-CoV-2 into cells	Orio et al., (2017)
3D tissue models	CBD extracts	Anti-inflammatory	13 high CBD / low THC lines were identified that modulate TMPRSS2 and ACE2 levels to reduce the virus effect	Wang et al., (2020)
Lung epithelial cell model	CBD, THCV, CBG, and multiple terpenes	Anti-inflammatory;Pro-inflammatory	Decreased levels of interleukin (IL) -6 and -8, Decrease in lung inflammation, Increased IL-6 and IL-8 expression in macrophages	Anil et al., (2021)
in vitro and in silico approaches	α-ketoamide, THCA,THC, CBN, CBD, CBDA	Antivirals;Pro-inflammatory	Using THC and CBD in combination with other drugs in the treatment of COVID-19 patients	Raj et al., (2021)
A549 humanlung carcinoma cells	CBD and 7-OH-CBD	Prevention of SARS-CoV-2 replication	SARS-CoV-2 replication blocked in lung epithelial cells	Nguyen et al., (2021)
ARDS inducedby poly(I:C)	CBD	Anti-inflammatory	Improvement of clinical symptoms of ARDS and decrease in proinflammatory cytokine level caused by Poly I: C	Khodadadi et al., (2021)
ARDS inducedby poly(I:C)	CBD	Regulation of apelin level in the blood,Anti-inflammatory	Increase in blood apelin expression	Salles et al., (2020)
Human lung fibroblasts	NT-VRL-1 with CBD	Amplified antiviral effect	Preventative treatment directly to the lungs	Chatow et al., (2021)
EpiDermFT model	Cannabis extracts	Pro-inflammatory, Anti-inflammatory,Anti-fibrotic	Inhibition of COX-2 and IL-6 levels in WI-38 lung fibroblasts	Kovalchuk et al., (2021)

In another preclinical study revealing the positive potential of CBD for the treatment of COVID-19, polycytidylic acid poly (I: C), a synthetic analogue of viral double-stranded RNA, was used in mice to induce ARDS (Salles et al., 2020). As a result of CBD administration, parallel to the improvement in the lung structure, T cells and neutrophils reached their normal levels, along with an increase in the level of apelin compared to the control. As plant essential oils, terpenes are the second most abundant metabolites in hemp after cannabinoids. Glycyrrhizin, which is also abundant in cannabis plants, has been shown to have antiviral effects against various SARS-CoV species (Cinat et al., 2003). In this context, in a recent study on in vitro evaluation of a mixture of terpene and cannabidiol activity against human coronavirus E229i, the combination of NT-VRL-1 (Code-name of terpene-based formulation) with CBD potentiated the antiviral effect (Chatow et al., 2021). In parallel, in a recent study it has been hypothesized that seven novel*C. sativa* extracts reduce the expression of pro-inflammatory cytokines and pathways related to fibrosis and inflammation (Kovalchuk et al., 2021), anti-TNFα, and anti-IL-6 cannabis extracts have been shown to contribute to existing anti-inflammatory regimens in treating COVID-19. A previous report showed that a combination of major terpenes and CBD works twice more than corticosteroids in treating COVID-19, albeit the final report of this study is still unpublished.^3^ In another recent study, cannabidiol has also been proven to be a stronger antiviral than reference drugs such as lopinavir, chloroquinone, and remdesivir (Raj et al., 2021). As a result of this study, it has been shown that cannabidiol in combination can be used to treat SARS-CoV-2 patients. In a very recent study with the administration of CBD and 7-OH-CBD to human lung carcinoma cells, SARS-CoV-2 infection in its early stages was likely blocked by 7-OH-CBD, the same metabolite included in the CBD treatment of epilepsy, and, thus, it has been suggested that lower SARS-CoV-2 infection occurs (Nguyen et al., 2021).

^3^Forbes (2020). New Research Suggests Terpenes And CBD Work 2X’s Better For Covid-19 Inflammation Than Corticosteroid. Website https://www.forbes.com/sites/emilyearlenbaugh [accessed 04 May 2021]

In brief, preclinical studies show that the administration of CBD through therapeutic interventions has antiviral, pro-inflammatory, and anti-inflammatory effects on organs targeted by the coronavirus; these may have been achieved mainly from the actions of CBD itself, or by synergistic chemicals such as terpenes that contribute to this action. Although the exact mechanisms have not been fully elucidated, several studies have shown that the endocannabinoid system; the involvement of endocannabinoids, cannabinoid receptors, and cannabinoid enzymes is described above.

### 3.11. Clinical evidence of cannabinoid efficacy

As of May 15, 2021, in the ClinicalTrials.gov database, there are nine clinical trials (two not yet recruiting, two active, not recruiting, and five recruiting) on CBD use in the context of COVID.^4^ As these studies will take some time to be completed, numerous other clinical trials have been reported in the ClinicalTrials.gov database indicating that CBD, THC, or both, or their synthetic derivatives, could be used in the prevention of COVID 19 (653 items regarding cannabinoids; 311 studies were signed as completed, 30 terminated, 151 recruiting, 56 not yet recruiting and 105 of them withdrawn or unknown or enrolling by invitation).

ClinicalTrials.gov (2021). U.S. National Library of Medicine. COVID-19 Information Dashboard [online]. Website https://www.ncbi.nlm.nih.gov/sars-cov-2/[accessed 15 May 2021]

In the database, contrary to preclinical evidence of cannabinoid efficacy for COVID-19 (Table), a large number of clinical trials are underway that will show their promising effects in the near future. However, cannabinoids are currently used as anxiolytic, relaxing, and anti-inflammatory therapeutic agent that can help in cases of epilepsy, schizophrenia, multiple sclerosis, depression, or chronic pain. Below, we discuss the details of 5 clinical studies reported in the database on 3 different pathologies (reducing pain, seizures, and the spasticity associated with multiple sclerosis, and fighting seizures of epilepsy in which cannabinoids are used most. There are seven studies dedicated to using the cannabinoids such as GW-1000-02 (THC) or CBD or Drug: Nabiximols (Sativex) (cannabis extract containing THC+CBD) in patients with pain-related symptoms, including the study entitled “Sativex for relieving persistent pain in participants with advanced cancer” (daily doses of 100 microliters (μL) oromucosal spray (2.5 mg CBD and 2.7 mg THC) in the evening and morning, up to a maximum of 10 sprays per day, for 5 weeks) and the study entitled “A two-part study of Sativex oromucosal spray for relieving uncontrolled persistent pain in patients with advanced cancer” (Nabiximols oromucosal spray contained CBD (25 mg/mL) and THC (27 mg/mL, for 5 weeks). Forty-eight studies (21 completed) are dedicated to the use of cannabinoids mostly cannabidiol or its derivatives such as Epidiolex (formerly, GWP42003-P) in patients with childhood epilepsy, including the study entitled “Antiepileptic efficacy study of GWP42003-P in children and young adults with dravet syndrome (GWPCARE1)” (A daily dose of 20 mg/kg/day for 11 days and this dose should be used for 12 weeks) and the study entitled “Efficacy and safety of GWP42003-P for seizures associated with Lennox-Gastaut syndrome in children and adults” with a daily dose of 10 and 20 mg (mg) per kilogram (kg) per day (mg/kg/day). Twenty-five studies (17 completed) are dedicated to the use of drug: Sativex, drug: GW-1000-02 or cannabis extract containing THC+CBD in the spasticity and seizures associated with multiple sclerosis, including the clinical study entitled “Sativex versus placebo when added to existing treatment for central neuropathic pain in MS” (with a daily dose of 8-12 sprays, each actuation contains 2.7 mg THC and 2.5 mg CBD). These clinical studies demonstrating the potential therapeutic properties of cannabinoids indicate that CBD or THC or their synthetic derivatives could also be used in the treatment of COVID-19 related disorders. Although clinical trials on COVID-19 are in their infancy, the following clinical studies have demonstrated that there are therapeutic effects of CBD on chronic pain (Capano et al., 2020), respiratory illnesses (Abdallah et al., 2018), inflammation-related disorders (Couch et al., 2019), anxiolytic properties (Zuardi et al., 2017), anxiety and sleep (Shannon et al., 2019), chronic schizophrenia (Boggs et al., 2018). Therefore, CBD alone or in combination with THC can be used as an adjuvant therapy to improve the quality of life of patients with COVID-19 and even to reduce the stress symptoms that may develop after recovery.

## 4. Conclusion and future perspectives

Of the many corona virus strains (SARS, MERS and COVID 19) observed over the last nearly two decades, COVID-19 has been the deadliest Coronavirus pandemic in human history. The duration and effectiveness of vaccines against SARS-CoV-2 cannot be predicted yet. In this context, the use of cannabis cannabinoids, especially CBD alone as a non-psychoactive cannabinoid or in combination with THC or terpenes, to limit or stop the severity of the disease based on reported incentive preclinical studies, in addition to existing vaccines, should be carefully studied to achieve protection against COVID-19. However, more evidence is needed for the routine use of cannabinoids and particularly non-psychoactive CBD in the treatment of COVID-19. Therefore, we hope that our hypothesis, supported by a large number of preclinical evidence and continuous clinical trial results, will inspire further targeted clinic studies to offer natural treatment options or the development of a broad spectrum medication for coronaviruses including SARS-CoV-2 responsible for COVID-19.

**Conflict of interest**

The authors declare that there is no conflict of interest.

**Acknowledgment**

We would like to thank Mr. İbrahim TÜNİK for his invaluable linguistic corrections.
